# Risk Factors and Nomogram Model for Hepatocellular Carcinoma Development in Chronic Hepatitis B Patients with Low-Level Viremia

**DOI:** 10.7150/ijms.95861

**Published:** 2024-06-17

**Authors:** Yu-Ching Chen, Chun-Chi Yang, Hsing-Tao Kuo, Ming-Jen Sheu, I-Che Feng, Chung-Feng Liu, Chi-Shu Sun, Su-Hung Wang, Cheng-Yi Lin, Chi-Hsing Chen, Sheng-Hsiang Lin

**Affiliations:** 1 Division of Gastroenterology and Hepatology, Department of Internal Medicine, Chi Mei Medical Center, Yongkang District, Tainan, Taiwan.; 2 Department of Internal Medicine, Chi Mei Medical Center, Yongkang District, Tainan, Taiwan.; 3 Department of Medical Research, Chi Mei Medical Center, Tainan, Taiwan.; 4 Institute of Clinical Medicine, College of Medicine, National Cheng Kung University, Tainan, Taiwan.; 5 Department of Public Health, College of Medicine, National Cheng Kung University, Tainan, Taiwan.; 6 Biostatistics Consulting Center, National Cheng Kung University Hospital, College of Medicine, National Cheng Kung University, Tainan, Taiwan.

**Keywords:** HBV, Low viral load, HCC, nomogram, Taiwan

## Abstract

**Background and aim:** Patients with chronic hepatitis B patients (CHB) with low-level viremia (LLV) are not necessarily at low risk of developing hepatocellular carcinoma (HCC). The question of whether CHB patients with LLV require immediate antiviral agent (AVT) or long-term AVT remains controversial. The study aims to investigate the risk of HCC development and the risk factors in CHB patients with LLV and construct a nomogram model predicting the risk of HCC.

**Methods:** We conducted a retrospective cohort study that enrolled 16,895 CHB patients from January 2008 to December 2020. The patients were divided into three groups for comparison: the LLV group, maintained virological response (MVR) group and HBV-DNA>2000 group. The cumulative incidence of progression to HCC was assessed. Cox regression analysis was performed to determine the final risk factors, and a nomogram model was constructed. The 10-fold Cross-Validation method was utilized for internal validation.

**Results:** A total of 408 new cases of HCC occurred during the average follow-up period of 5.78 years. The 3, 5, and 10-year cumulative HCC risks in the LLV group were 3.56%, 4.96%, and 9.51%, respectively. There was a significant difference in the cumulative risk of HCC between the HBV-DNA level > 2000 IU/mL and LLV groups (p = 0.049). Independent risk factors for HCC development in LLV group included male gender, age, presence of cirrhosis, and platelets count. The Area Under the Curve (AUC) values for the 3-year and 5-year prediction from our HCC risk prediction model were 0.75 and 0.76, respectively.

**Conclusion:** Patients with LLV and MVR are still at risk for developing HCC. The nomogram established for CHB patient with LLV, incorporating identified significant risk factors, serves as an effective tool for predicting HCC-free outcomes. This nomogram model provides valuable information for determining appropriate surveillance strategies and prescribing AVT.

## Introduction

Hepatitis B virus (HBV) infection is a major cause of chronic liver disease, liver cirrhosis, and hepatocellular carcinoma (HCC). The high replication of HBV and the development of cirrhosis increase the risk of developing liver cancer 1. HBV infection poses a significant global public health threat, affecting over 257 million people worldwide. The prevalence of HBV infection exhibits considerable heterogeneity globally, with Taiwan being particularly endemic for chronic hepatitis B (CHB). According to the World Health Organization (WHO) statistics, over 88,000 people die annually due to HBV infection 2,3. HBV is the primary cause of both the incidence and mortality of liver cancer worldwide, with cirrhosis being a major risk factor for HCC 4. Despite recent advancements in anticancer drugs improving the efficacy against advanced HCC, the severity of cirrhosis still hinders effective treatment for HCC 5. In 2015, it was estimated that there were 854,000 new cases of liver cancer and 810,000 deaths globally 6. HCC continues to contribute to the increasing global disease burden, and according to the WHO's annual estimates, by 2030, over one million liver disease patients are projected to die from liver cancer 7.

Oral nucleos(t)ide analogs (NUCs) are the primary form of global antiviral therapy (AVT) for treating HBV. AVT can significantly impact the natural course of CHB, and long-term AVT effectively suppresses HBV replication, reducing the occurrence of complications such as liver disease progression, cirrhosis, and HCC 1,8-10. However, under the treatment of potent NUCs with high resistance barriers, only 5%-10% of CHB patients achieve functional cure, defined as the disappearance of hepatitis B surface antigen (HBsAg) and undetectable serum HBV-DNA 10. The majority of CHB patients maintain low viral loads with serum HBV-DNA levels either below 2,000 IU/mL or detectable but with low viral quantities (globally recommended detection limit of 20 IU/mL), a condition known as low-level viremia (LLV). Patients with LLV remain exposed to the risk of developing cirrhosis or HCC.

Several studies have reported an association between CHB patients presented lower, yet detectable LLV (HBV-DNA levels of 12-1,999 IU/mL) and a higher risk of HCC compared to those with undetectable viral loads 8,11-13. However, some studies have reported contradictory results. Cho et al. 14, Lee et al. 15, and Huang et al. 16 found no significant difference in the risk of HCC between patients with undetectable viral loads and those with LLV. There is still ongoing disagreement regarding whether CHB patients with LLV should undergo immediate AVT or if, similar to patients with cirrhosis, long-term AVT is necessary. Insufficient clinical data currently exist to determine whether LLV is a benign disease state. Based on published research findings, the risk of HCC in patients with LLV is not low. Consequently, there is a need for further evaluation of the dynamic interplay between LLV, the extent of fibrosis, and the development of HCC.

To date, various prediction models for HCC risk have been extensively developed 17, offering clinical practitioners assistance in assessing treatment and follow-up. Currently, there is no dedicated HCC prediction model specifically designed for the LLV population with potential HCC risk. Therefore, this study aims to investigate the risk factors and prognosis analysis for HCC occurrence in CHB patients with accompanying LLV. Additionally, the study aims to establish an HCC risk prediction nomogram model for individuals with LLV.

## Materials and Methods

### Study population

This retrospective cohort study was conducted with CHB patients at three medical institutions of Chi Mei hospital (Tainan, Taiwan) including Yongkang Chi Mei Medical Center, Liuying branch, and Jiali branch, from January 2008 to December 2020. Patients who meet the following criteria will be included: (1) age ≥ 20 years; (2) Serum HBsAg positive with and without detected HBV-DNA. The exclusion criteria were as follows: (1) with a history of HCC or liver decompensation; (2) with a history of liver transplantation; (3) co-infection with other hepatitis virus infection or human immunodeficiency virus (human immunodeficiency virus, HIV) infection; (4) previous history or ongoing hepatic decompensation; (5) chemotherapy or immunosuppressant treatment use; (6) other major diseases; (7) liver function or related data were incomplete.

During the follow-up period, according to the serum HBV-DNA level, patients will be divided into LLV group, Maintained viral response (MVR) group and HBV-DNA>2000 group. Patients in the LLV group were defined as serum HBV-DNA ≥20 IU/mL but ≤2,000 IU/mL at least once during the follow-up period. According to the recommendations of international clinical guidelines, the MVR group, as the NUCs treatment target, is defined as including patients with persistently undetectable residual HBV-DNA (<20 IU/mL) 18,13,14. During the follow-up period, AVT was commenced if the reimbursement criteria established by the Taiwan National Health Insurance (NHI) were fulfilled (see Supplementary Table 1) 19.

The study protocol was performed in accordance with the principles of the Declaration of Helsinki, and it was reviewed and approved by the institutional review board (IRB) at Chi Mei Medical Center as number 11104-015. The study is based on the retrospective analysis design of existing de-identified clinical data, the informed patient consent was waived.

### Clinical assessment and study end points

CHB was defined as HBsAg positivity for more than six months or once detected HBV DNA 20. The diagnosis of cirrhosis was defined by the results of liver biopsy, ultrasound, elastographic evidence, cross-sectional image study (including computed tomography (CT) or magnetic resonance imaging (MRI)), or advanced fibrosis combined with other signs of portal hypertension like esophageal-gastric varices formation 21. The ultrasound examination was conducted by experienced doctors and followed specific criteria, including assessing for nodular liver surface, caudate lobe hypertrophy, and splenomegaly, to diagnose cirrhosis. Additionally, clinical physician would consider platelet count, bilirubin levels, and albumin levels as supplementary evidence. HCC was diagnosed based on histological or radiological evidence and was further assessed by clinical experienced physicians 22. MVR was defined as serum HBV DNA undetectable containing <20 IU/mL at baseline despite HBsAg being positive. LLV was defined as detectable HBV DNA between 20 to 2000 at baseline.

Demographic characteristics and baseline data, including demographics, comorbidity (including diabetes mellitus and history of other malignancy), blood routine examination, hepatic function, renal function, coagulation tests, sero-marker of hepatitis B including HBsAg, HBeAg, HBV DNA, and alpha-fetoprotein (AFP), were recorded at the first time we decided to enroll the particular. We also recorded AVT which uses NUCs, including adefovir, entecavir, lamivudine, telbivudine, tenofovir, and tenofovir alafenamide 23.

Patients were under surveillance with routine laboratory tests, serum AFP and radiological examination every 3 to 6 months. The primary outcome was the occurrence of HCC during follow-up. The degree of liver fibrosis is measured by FIB-4 using non-invasive and inexpensive equipment, based on, which is calculated with the formula to observe the changes in liver fibrosis during the follow-up period. FIB-4 >1.45 indicates obvious fibrosis, and >3.25 indicates cirrhosis 24-26.

### Statistical analysis

Data are expressed as means ± SDs or numbers (%), as appropriate. Continuous variables and categorical variables were compared by Student's t-test (or Mann-Whitney U test) and χ 2 test (or Fisher exact test). Differences in the cumulative incidence of HCC among LLV, MVR, and HBV-DNA ≥ 2000 IU/mL were assessed using the Kaplan-Meier method and compared using the log-rank test. The cutoff points were determined based on the normal range for the alanine aminotransferase (ALT), AST, total bilirubin, serum albumin, platelet count, creatinine (Cr), and international normalized ratio (INR). The cut-off values of AFP were 400 ng/mL, and FIB-4 scores. The HBV DNA level was divided into <20, 20-2000, and over 2000 IU/mL.

After evaluating the importance of all independent variables through the univariate Cox regression model, significant variables (P < 0.05) were extracted and included in multivariate models for further analysis, presented with hazard ratios (HRs) and 95% confidence intervals (CIs). Multivariate Cox regression analysis was then employed to identify the final risk factors (P < 0.05) for establishing a nomogram to predict the risk of HCC. The score for each key variable is determined through linear transformation based on the absolute value of the β coefficient calculated in the multivariate Cox regression analysis. The total score, derived from summing the scores assigned to each key variable, represents the score of the prediction model 27-29.

The discriminative performance of the prediction model was evaluated by the area under the curve (AUC) of the ROC curve 30,31. The time cutoffs are set at 3 years (1095 days) and 5 years (1825 days), along with the corresponding AUC results at different time points and summarizing the overall outcomes during the follow-up period (Integrated time-dependent AUC). The internal validation through the 10-fold Cross-Validation method. The 10-fold Cross-Validation sample is divided into ten equal parts from the original sample, one-tenth of the sample is used as the validation group, and the remaining nine equal samples are used to train the prediction model 27,28,32-35. All statistical analyses were performed using SAS 9.4 with R 4.1.3. Statistical significance was defined as a two-tailed P < 0.05.

## Results

### Baseline clinical characteristics of the study population

A total of 16,895 patients were finally eligible for inclusion in this study, and men accounted for 59% of all patients (n=10,019). HCC development was observed in 846 (5.0%) patients during the follow-up period. The clinical characteristics and laboratory data of the patients are presented in Table 1, as well as those with HCC (n=846) and those without HCC (n=16,049). Among the patients with HCC, 663 (78.37%) were male, with an average age of 58.19 years old, 208 (24.59%) were diabetic, 376 (44.44%) had a history of liver cirrhosis, and 103 (18.29%) were HBeAg positive. Out of the total 646 (92.95%) patients, the liver fibrosis indicator (FIB-4) was categorized as F0, indicating the absence of liver fibrosis. A total of 1351 people were enrolled in LLV group, of whom 59.73% were male (n=807). During the follow-up period, 79 patients developed HCC. Among the HCC patients, 59 (74.68%) were male, with an average age of 57.62 years, 20 (25.32%) were diabetic, 37 (46.84%) had a history of liver cirrhosis, and 8 (11.94%) had seropositive of HBeAg. Out of the total HCC patients in LLV group, 68 (93.15%) patients were categorized as F0 by FIB-4, indicating the absence of liver fibrosis.

### Risk of HCC development according to HBV-DNA level

A total of 408 new cases of liver cancer occurred during the average follow-up period of 5.78 years (interquartile range 0.0027-10 years). During the follow-up period, the 3, 5 and 10-year cumulative risks of HCC in the LLV group were 3.56%, 4.96% and 9.51% respectively, and the 3, 5 and 10-year cumulative risks of HCC in the MVR group were 3.64%, 4.98% and 10.54% respectively, while the 3, 5 and 10-year cumulative risks of HCC in the HBV-DNA>2000 IU/mL group were 4.38%, 6.68% and 12.37% respectively (Supplementary Table 2).

**Figure 1:**
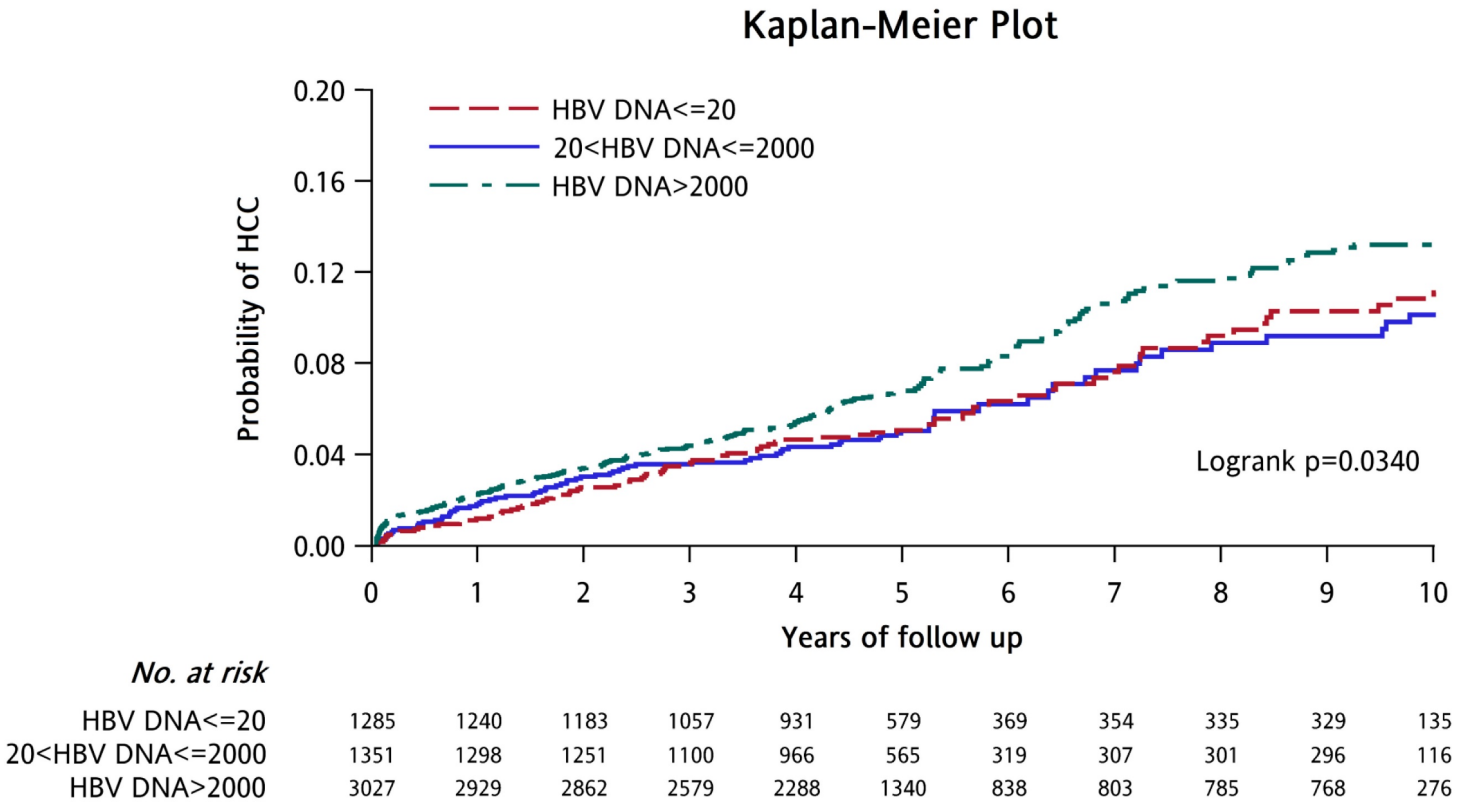
** Cumulative incidence of hepatocellular carcinoma according to HBV-DNA levels.** Abbreviations: HCC: hepatocellular carcinoma. HBV: Hepatitis B virus.

During the 10-year follow-up period, it was observed that there were significant differences in the cumulative risk of HCC and the incidence of HCC-free (p=0.034) between the LLV group, the MVR group, and the HBV-DNA>2000 group (Figure 1). The cumulative risk of HCC among the three groups was compared, and a significant difference was observed between the HBV-DNA level>2000 IU/mL and LLV groups (p=0.049), which is consistent with the results of past studies. When HBV-DNA level>2000 IU/mL, CHB patients are at a higher risk of developing HCC 36-39. However, there was no significant difference between the LLV group and the MVR group (p=0.970) and between the HBV-DNA>2000 group and the MVR group (p=0.078) (Supplementary Table 2). Our results indicate that patients with LLV and those with MVR face a comparable risk of developing HCC during the 10-year follow-up period.

### Independent risk factors associated with the development of HCC

Table 2 presents the overall multivariate Cox regression analysis to identify risk factors independently associated with the development of HCC. In terms of HCC development, male (adjusted hazard ratio [aHR]: 1.99, 95% CI: 1.64-2.41; P<0.001), age (aHR: 1.05, 95% CI: 1.04-1.05; P<0.001), Those with other cancers aHR: 1.24, 95% CI: 1.06-1.46; P=0.009), those with liver cirrhosis (aHR: 2.86, 95% CI: 2.37-3.43; P<0.001), Platelet (aHR: 1.63, 95% CI: 1.36-1.95; P<0.001), AST (aHR: 2.09, 95% CI: 1.62-2.69; P<0.001), and AFP (aHR: 11.72, 95% CI: 8.16-16.84; P<0.001) are independent significant predictors. In univariate analysis, sex, age, presence of cirrhosis, eGFR, abnormal level of platelet count and AST, and FIB-4 score over 1.3 were significantly associated with HCC occurrence in patient with LLV (all P < 0.05). These significant factors were included in a subsequent multivariate Cox regression analysis and suggesting male (aHR: 1.78, 95% CI: 1.04-3.04; P=0.036), age (aHR: 1.05, 95% CI: 1.03-1.07; P<0.001), presence of cirrhosis (aHR: 2.01, 95% CI: 1.17-3.45; P=0.012) and Platelet (aHR: 2.19, 95% CI: 1.26-3.79; P=0.005) were independently significant predictor factor for HCC occurrence.

### Construction of HCC risk scoring system and prediction model

Through multivariate Cox regression analysis, we identified key independent risk factors associated with the occurrence of HCC in patients with LLV. Subsequently, we constructed an HCC risk prediction model (see Table 2). The weighted values for each crucial variable were determined based on the β coefficients calculated through multivariate Cox regression analysis. This risk scoring model includes sex, age, concurrent presence of other cancers, presence of liver cirrhosis, platelet count, and AST.

**Figure 2:**
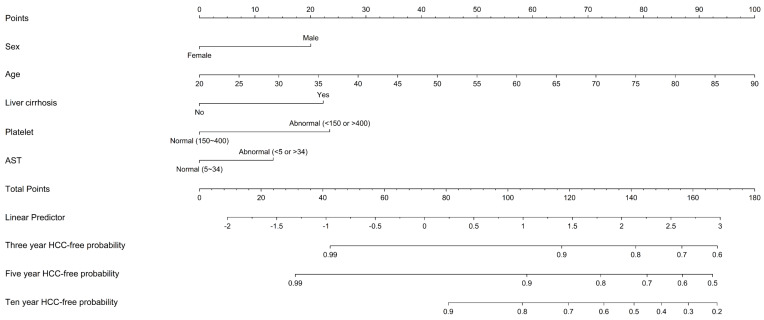
** Nomogram predicting the risk of hepatocellular carcinoma in patients with chronic hepatitis B.** Abbreviations: HCC: hepatocellular carcinoma. HBV: Hepatitis B virus. AST: aspartate aminotransferase.

The cumulative score was computed based on the individual scores derived from the nomogram. Figure 2 illustrates a nomogram presenting a predictive model for assessing the risk of HCC in patients within the LLV group. Each variable is allocated a specific point on the upper axis by extending a line upwards. The total of these points corresponds to a position on the total points axis, and a line is drawn downward to intersect with the probability axis, thereby establishing the probability of HCC. The total sum of points assigned to each key variable ranges from 9 to 165 points. The table for score comparison, aiding in the prediction of HCC risk, can be found in Supplementary Table 3.

### Predictive performance of the HCC risk prediction model

**Figure 3:**
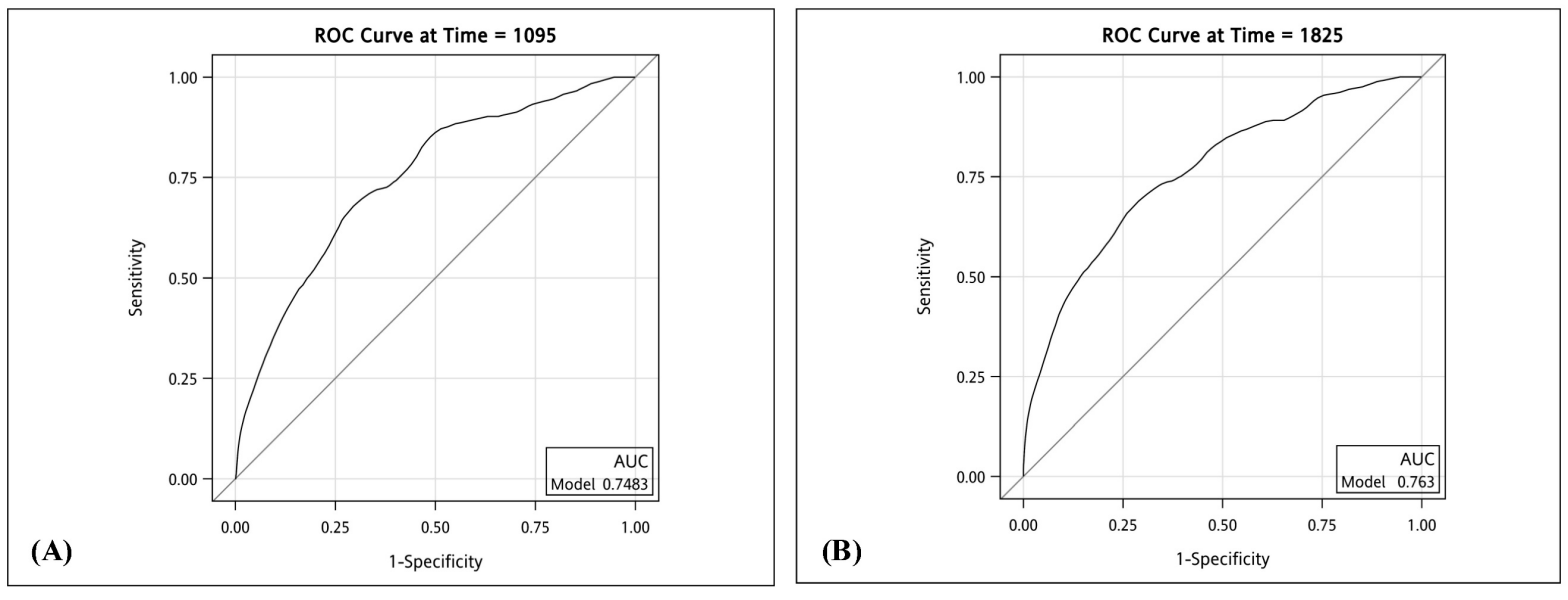
** (A) Time-dependent AUC of three years HCC-free in patients with chronic hepatitis B. (B) Time-dependent AUC of five years HCC-free in patients with chronic hepatitis B.** Abbreviations: ROC: receiver operating characteristic. AUC: area under the ROC curve. HCC: hepatocellular carcinoma.

**Figure 4:**
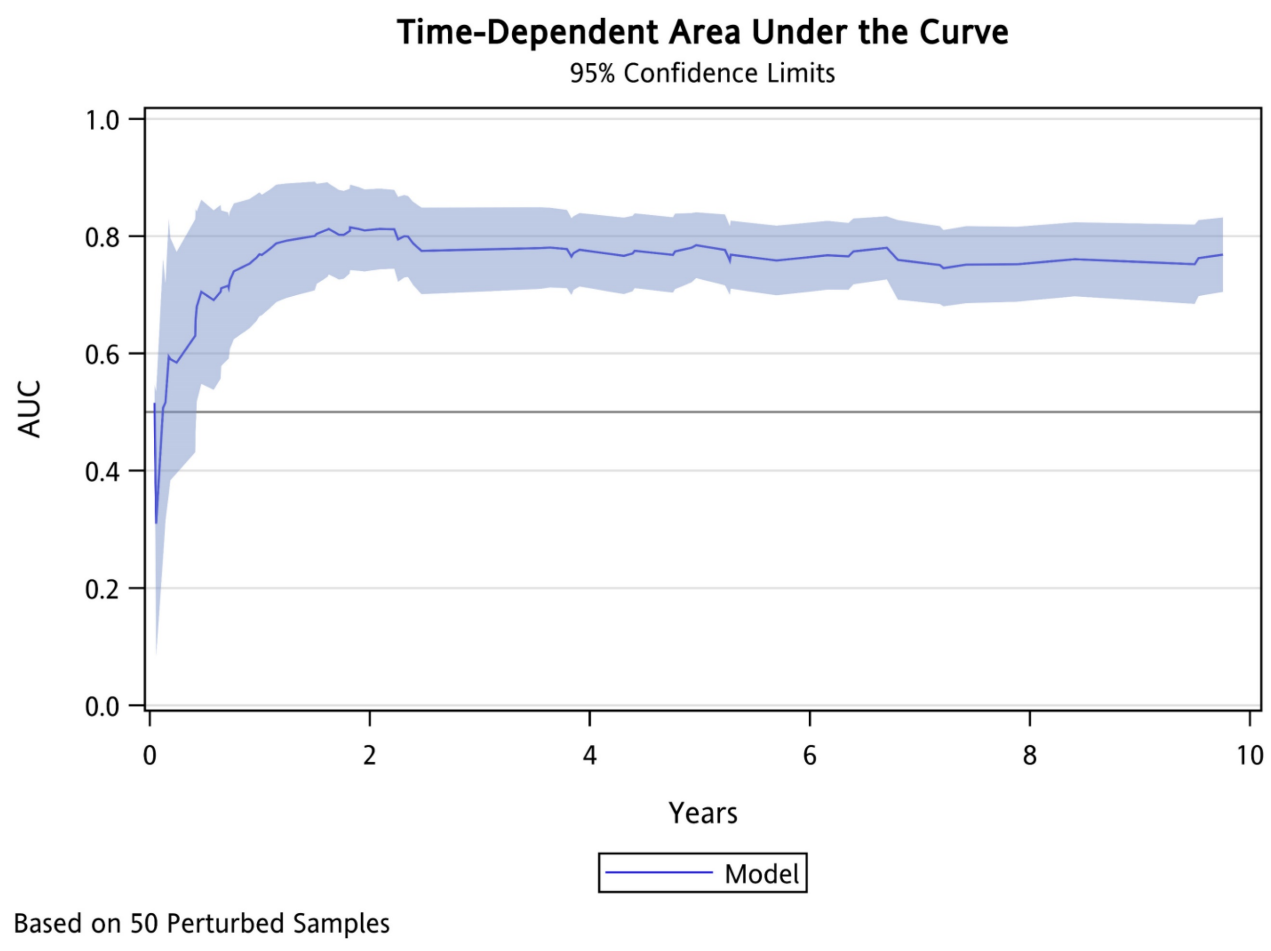
** Integrated time-dependent AUC to predict HCC-free in patients with chronic hepatitis B.** Abbreviations: AUC: area under the ROC curve. HCC: hepatocellular carcinoma.

We employed ROC curves to illustrate and evaluate the discriminative performance of the 3-year and 5-year HCC-free risk prediction models in patients with LLV. The AUCs for the 3-year and 5-year models were 0.75 (sensitivity 0.71, specificity 0.67) and 0.76 (sensitivity 0.73, specificity 0.66), respectively (see Figure 3). The HCC risk prediction models underwent validation using the 10-fold cross-validation method. In the validation group, the AUC values for the 3-year and 5-year predictions were 0.73 (sensitivity 0.82, specificity 0.64) and 0.75 (sensitivity 0.77, specificity 0.66), respectively. These results suggest a strong predictive ability of the model. Figure 4 illustrates the integrated time-dependent AUC curve for predicting the absence of HCC as 0.75. The integrated time-dependent AUC, verified through the 10-fold cross-validation method, is 0.74.

## Discussion

This study is the first article to develop a risk prediction model for HCC for LLV patients. Furthermore, the present study utilized clinical routine parameters, making the risk prediction model convenient and accurate. This approach enhances its applicability in clinical practice, offering a practical tool for assessing the risk of HCC in patients with LLV. The model can serve as a valuable reference for future research endeavors, providing a foundation for ongoing investigations and contributing to the advancement of our understanding of HCC risk factors in CHB patient with LLV. Finally, by capturing the real-world progression of CHB over an extended period, the findings contribute valuable insights to the understanding of the natural course of CHB with LLV and its implications for HCC risk.

It is well known that active replication and a high viral load of HBV are more likely to lead to a poor outcome or hepatitis-related complications 40, 41. A study conducted by Yuan et al. in Hong Kong revealed that the major factor contributing to the development of cirrhotic complications was viral load. However, cirrhotic complications continued to develop in a quarter of anti-HBe patients with HBV-DNA levels below 10^4 copies/mL 42. The long-term clinical impact of CHB patients with LLV, defined as HBV-DNA < 2,000 IU/mL), remains unclear based on retrospective analysis in past studies. A multicenter study conducted by Sinn et al. in Korea revealed that the 5-year cumulative HCC incidence rate was 13.9% for cirrhotic patients with LLV who were treatment naïve. This rate was higher compared to the HCC incidence rate of patients with undetected HBV-DNA (8.2%) 12.

Recently, several studies have examined the association between LLV status and increased HCC risk in patients with CHB receiving AVT compared to those with MVR 8,11-13. The results of this study revealed that the 3-year and 5-year cumulative HCC incidence rates for MVR patients were 3.2% and 7.5%, respectively, which were lower than those for LLV patients (6.2% and 14.3% at 3 and 5 years, respectively; P = 0.016). Furthermore, when stratified by the presence of cirrhosis, individuals with LLV status had a significantly higher risk of developing HCC compared to those with MVR (5-year cumulative HCC incidence rates of 23.4% vs. 10.3%, respectively; P = 0.001) 8. The relationship between LLV and HCC development was even more significant in cirrhotic patients (adjusted HR = 2.20, 95% CI = 1.34-3.60; P = 0.002), with LLV identified as an independent risk factor for HCC development in cirrhotic patients. In individuals without cirrhosis, there was no significant difference in the cumulative incidence rates of HCC between MVR and LLV groups, but the LLV groups still had a relatively higher risk. (5-year HCC incidence rates of 4.0% vs. 6.9%, respectively; P = 0.44) 8.

In our study, we identified the risk factors associated with the occurrence of HCC in CHB patients with LLV. These risk factors included male sex, age, the presence of liver cirrhosis, the use of Baraclude or Telbivudine (both are AVT), platelet count, eGFR, and AST levels. Additionally, ALT and FIB-4 scores showed a borderline association. The presence of cirrhosis is the most important risk factor for HCC development (aHR: 2.86, 95% CI: 2.37-3.43; P < 0.001), which is consistent with findings from other studies focusing on the risk of HCC development 8,12. Therefore, we are convinced that CHB patients with LLV and concurrent cirrhosis should be closely monitored, and early initiation of antiviral treatment may be advisable to reduce the risk of HCC occurrence. Following adjustment through multivariate Cox regression analysis, some key predictive factors associated with the HCC development in LLV patients, including male sex, age, presence of cirrhosis, and platelet count. Notably, these predicting factors determined by multivariate Cox regression analysis are common laboratory parameters widely used in clinical practice. Consequently, the nomogram established in our study for predicting the risk of HCC development in CHB patient with LLV demonstrates good accuracy and practical convenience for clinical application.

Several international guidelines provide recommendations for the clinical practice of CHB 8,9. Among these, guidelines from the American Association for the Study of the Liver (AASLD), the European Association for the Study of the Liver (EASL), and the Asia-Pacific Association for the Study of the Liver (APASL) are widely utilized. For compensated cirrhosis patients, both AASLD and EASL guidelines advocate treatment for cirrhotic patients with LLV (<2000 IU/mL), whereas APASL guideline does not. In Taiwan, CHB patients can initiated AVT with reimbursement from the Taiwan NHI if the specific reimbursement criteria are met. These criteria include (1) HBeAg-positive CHB patient with abnormal ALT levels combined HBV-DNA > 20,000 IU/mL, (2) HBeAg-negative CHB patient with abnormal ALT levels combined HBV-DNA > 2,000 IU/mL, (3) CHB patient with cirrhosis concomitant with obvious evidence of portal hypertension, and (4) meet the criteria of acute on chronic liver failure. Therefore, CHB patient with LLV in Taiwan may face challenges in obtaining reimbursement for NUCs, even though they carry a significant risk of developing cirrhosis and HCC. To bridge this gap, we developed and validated a simple nomogram to estimate the risk of HCC in CHB patients with LLV. The nomogram, based on readily available predictors, demonstrated effective predictive performance.

The results of present study revealed no statistically significant difference in the risk of HCC between patients with LLV status during the follow-up period and patients in the MVR group whose HBV-DNA remained continuously undetectable (<20 IU/mL) (P=0.970). The risk is comparable and aligns with the findings of three recent retrospective cohort studies 14-16. Despite this, four earlier studies observed a higher incidence of HCC in patients with LLV compared to patients with MVR 8,11-13. There remained disagreement on whether CHB patients with LLV status necessitate immediate AVT or long-term AVT similar to patients with cirrhosis. Additionally, there is insufficient clinical data to determine whether LLV presents a benign disease state. Further randomized controlled trials are necessary to propose more cost-effective and accurate clinical strategies.

This study has several strengths and, to the best of our knowledge, represents the first long-term analysis comprehensively assessing simultaneously the risk of developing HCC in patients with LLV, MVR, and HBV-DNA >2000, with or without AVT. This study benefits from a large population sample and a sufficient number of events, with 408 (7.20%) cases of HCC and 607 (10.72%) deaths, providing robust statistical power. The extended follow-up period (average follow-up time of 5.78 years) enhances the reliability of the statistical results.

This study has some limitations. Firstly, in comparison to a prospective research design, the compliance of this study population with HCC monitoring may not be as meticulous. Additionally, the study did not conduct a direct comparison between patients who received AVT and those who did not. The decision not to compare these groups was influenced by the fact that patients receiving AVT had distinct baseline clinical characteristics compared to those who did not undergo AVT due to the criteria of NHI reimbursement. It is important to note that this study employed abdominal ultrasound for the diagnosis of liver cirrhosis, a method that may rely on the subjective judgment of individual physicians. Secondly, patients who received AVT initiated treatment at varying time points throughout the follow-up period. Over the course of treatment, issues such as adverse effects, drug resistance, and compromised renal function may lead to alterations in medication or relapses following treatment cessation, prompting a subsequent round of AVT. Owing to factors such as patients transitioning from the original treatment hospital or becoming lost to follow-up for unknown reasons, it is impossible to obtain detailed information on the AVT of the detailed case during the treatment period. Furthermore, most patients received treatment after HBV-DNA elevation, and during treatment, patients may experience HBV-DNA <2000 to the lowest detectable lower limit or undetectable viral load. These variations make it challenging to achieve a sufficient sample size and conduct a proper comparison. On the other hand, in Asia, including Taiwan, genotypes B and C are the predominant HBV genotypes, and both genotypes are associated with more severe liver disease and a higher risk of HCC development 15,38,43,44. Consequently, the findings of this study are specifically representative of the predominant Asian population.

Therefore, comprehending whether HCC occurs in CHB patients whose HBV-DNA declines to low viremia necessitates well-designed randomized controlled trials (RCTs) that effectively balance the risks of HCC and the benefits of AVT in patients. Despite this study's limitation, efforts were made to mitigate this issue through consistent results following various statistical adjustments. Additionally, the study benefited from a homogeneous study population within the region, a statistically reliable and adequate sample size and event rate, and an extended duration of follow-up. These strengths contribute to the robustness of the study's findings. Further exploration through well-designed prospective studies is needed to enhance our understanding of the topic, and more precise analyses could be conducted using methods such as mixed-effects models or generalized estimating equations (GEE) by incorporating repeated measurements. However, the results obtained in this study can still serve as valuable reference points, offering insights and guiding more in-depth research for future clinical and therapeutic strategies.

## Conclusion

In summary, this retrospective cohort study has identified significant risk factors for HCC in CHB patients with LLV, as well as the long-term outcomes associated with LLV status. While our findings suggest that the risk of HCC in patients with LLV is comparable to that in patients with MVR, persistent LLV status may still contribute to chronic low-grade inflammation and fibrotic changes, ultimately leading to HCC development. Timely detection of HCC and cirrhosis through regular surveillance is crucial for improving overall patient prognosis. Consequently, our study has developed an HCC risk prediction model tailored specifically to individuals with LLV status. The resulting nomogram provides a valuable tool for assessing individual risk and guiding the implementation of targeted HCC monitoring programs for LLV patients. This personalized approach can inform future treatment decisions, aid in the development of strategic interventions, and serve as a valuable reference for clinicians.

## Supplementary Material

Supplementary tables.

## Figures and Tables

**Table 1:** Clinical characteristics of patients with chronic hepatitis B and low level viremia group with or without hepatocellular carcinoma

	Total patients			LLV patients		
Variables	Non HCC (N=16049)	HCC (N=846)	p-value	Non HCC (N=1272)	HCC (N=79)	
	n (%) / mean ± SD	n (%) / mean ± SD		n (%) / mean ± SD	n (%) / mean ± SD	p-value
Male	9356 (58.30)	663 (78.37)	<0.001	748 (58.81)	59 (74.68)	0.008
Age	48.53 ± 13.48	58.19 ± 11.41	<0.001	49.86 ± 12.81	57.62 ± 11.76	<0.001
Comorbidity						
DM	2184 (13.61)	208 (24.59)	<0.001	206 (16.19)	20 (25.32)	0.051
Other cancer	6962 (43.38)	418 (49.41)	0.001	675 (53.07)	42 (53.16)	1.000
Liver cirrhosis	1324 (8.25)	376 (44.44)	<0.001	202 (15.88)	37 (46.84)	<0.001
Medication						
Adefovir dipivoxi (HEPSERA)	58 (0.36)	2 (0.24)	0.770	13 (1.02)	0 (0.00)	1.000
Entecavir Tab#1mg	470 (2.93)	36 (4.26)	0.035	80 (6.29)	9 (11.39)	0.123
Baraclude 0.5mg	1274 (7.94)	134 (15.84)	<0.001	197 (15.49)	21 (26.58)	0.015
ZEFFIX (Lamivudine)	403 (2.51)	23 (2.72)	0.793	69 (5.42)	2 (2.53)	0.431
Telbivudine (600mg)	349 (2.17)	32 (3.78)	0.003	60 (4.72)	10 (12.66)	0.006
Tenofovir 300mg (Viread)	1222 (7.61)	105 (12.41)	<0.001	165 (12.97)	12 (15.19)	0.693
Tenofovir alafenamide (Vemlidy)	95 (0.59)	3 (0.35)	0.490	14 (1.10)	0 (0.00)	1.000
Platelet						
Normal (150-400)	8572 (80.65)	352 (49.37)	<0.001	786 (76.46)	32 (43.84)	<0.001
Abnormal (<150 or >400)	2057 (19.35)	361 (50.63)		242 (23.54)	41 (56.16)	
PT						
Normal (9.4-12.5)	5384 (87.90)	534 (82.92)	<0.001	615 (86.26)	55 (83.33)	0.639
Abnormal (<9.4 or >12.5)	741 (12.10)	110 (17.08)		98 (13.74)	11 (16.67)	
Creatinine						
Normal (0.72-1.25)	8673 (71.39)	575 (72.33)	0.598	826 (71.45)	51 (67.11)	0.497
Abnormal (<0.72 or >1.25)	3476 (28.61)	220 (27.67)		330 (28.55)	25 (32.89)	
eGFR	79.21 ± 21.31	72.93 ± 23.00	<0.001	79.82 ± 22.79	74.39 ± 25.79	0.046
AST						
Normal (5-34)	9366 (64.54)	200 (24.84)	<0.001	741 (58.62)	26 (33.33)	<0.001
Abnormal (<5 or >34)	5145 (35.46)	605 (75.16)		523 (41.38)	52 (66.67)	
ALT						
Normal (2-40)	8778 (59.33)	276 (34.12)	<0.001	705 (55.64)	34 (43.59)	0.050
Abnormal (<2 or >40)	6018 (40.67)	533 (65.88)		562 (44.36)	44 (56.41)	
Bili Total						
Normal (0.2-1.2)	8054 (83.62)	557 (76.41)	<0.001	841 (79.87)	55 (72.37)	0.158
Abnormal (<0.2 or >1.2)	1578 (16.38)	172 (23.59)		212 (20.13)	21 (27.63)	
Albumin						
Normal (3.5-5.2)	4355 (79.79)	452 (73.14)	<0.001	487 (74.81)	43 (67.19)	0.239
Abnormal (<3.5 or >5.2)	1103 (20.21)	166 (26.86)		164 (25.19)	21 (32.81)	
HBsAg						
Negative	363 (12.83)	13 (8.78)	0.188	5 (2.70)	0 (0.00)	1.000
Positive	2466 (87.17)	135 (91.22)		180 (97.30)	12 (100.00)	
HBeAg						
Negative	8442 (83.92)	460 (81.71)	0.183	983 (89.53)	59 (88.06)	0.862
Positive	1617 (16.08)	103 (18.29)		115 (10.47)	8 (11.94)	
HBV_DNA						
≤ 20	1203 (22.89)	82 (20.10)	0.010	-	-	-
> 20- ≤ 2000	1272 (24.21)	79 (19.36)		1272 (100.00)	79 (100.00)	
> 2000	2780 (52.90)	247 (60.54)		-	-	
AFP						
< 400	12678 (99.79)	684 (93.06)	<0.001	1165 (99.49)	75 (98.68)	0.357
≥ 400	27 (0.21)	51 (6.94)		6 (0.51)	1 (1.32)	
FIB-4 scores						
F0 (<1.3)	10098 (98.04)	646 (92.95)	<0.001	1002 (97.76)	68 (93.15)	0.061
F1 (1.3-2.1)	128 (1.24)	34 (4.89)		18 (1.76)	5 (6.85)	
F2 (2.1-3.25)	46 (0.45)	12 (1.73)		4 (0.39)	0 (0.00)	
F3 & F4 (>3.25)	28 (0.27)	3 (0.43)		1 (0.10)	0 (0.00)	

Abbreviation: HCC: hepatocellular carcinoma. LLV: low level viremia. DM: diabetes mellitus. PT: prothrombin time. eGFR: estimated Glomerular filtration rate. AST: aspartate aminotransferase. ALT: alanine aminotransferase. Bili Total: Total Bilirubin. HBsAg: hepatitis B virus s antigen. HBeAg: hepatitis B virus e antigen. AFP: alpha-fetoprotein. FIB-4 score: Fibrosis-4 score.

**Table 2:** Independent risk factors for HCC in patients with chronic hepatitis B and low viremia group

	Total patients				LLV patients			
	Univariate analysis		Multivariable analysis		Univariate analysis		Multivariable analysis	
Variables	Crude HR (95% CI)	p-value	Adjusted HR (95% CI)	p-value	Crude HR (95% CI)	p-value	Adjusted HR (95% CI)	p-value
Male	2.51 (2.13-2.96)	<0.001	1.99 (1.64-2.41)	<0.001	1.98 (1.19-3.28)	0.008	1.78 (1.04-3.04)	0.036
Age	1.05 (1.05-1.06)	<0.001	1.05 (1.04-1.05)	<0.001	1.05 (1.03-1.07)	<0.001	1.05 (1.03-1.07)	<0.001
Comorbidity								
DM	1.98 (1.69-2.32)	<0.001	1.05 (0.88-1.26)	0.594	1.65 (0.99-2.74)	0.052		
Other cancer	1.26 (1.10-1.45)	0.001	1.24 (1.06-1.46)	0.009	0.99 (0.64-1.55)	0.978		
Liver cirrhosis	7.76 (6.78-8.89)	<0.001	2.86 (2.37-3.43)	<0.001	4.36 (2.80-6.79)	<0.001	2.01 (1.17-3.45)	0.012
Platelet								
Normal (150-400)	Ref.		Ref.		Ref.		Ref.	
Abnormal (<150 or >400)	4.10 (3.54-4.75)	<0.001	1.63 (1.36-1.95)	<0.001	3.86 (2.43-6.13)	<0.001	2.19 (1.26-3.79)	0.005
PT								
Normal (9.4-12.5)	Ref.				Ref.			
Abnormal (<9.4 or >12.5)	1.51 (1.23-1.85)	<0.001			1.29 (0.68-2.47)	0.440		
Creatinine								
Normal (0.72-1.25)	Ref.				Ref.			
Abnormal (<0.72 or >1.25)	1.00 (0.86-1.17)	0.966			1.33 (0.82-2.14)	0.249		
eGFR	0.99 (0.98-0.99)	<0.001	1.00 (1.00-1.00)	0.856	0.99 (0.98-1.00)	0.049	1.00 (0.99-1.01)	0.879
AST								
Normal (5-34)	Ref.		Ref.		Ref.		Ref.	
Abnormal (<5 or >34)	5.08 (4.33-5.96)	<0.001	2.09 (1.62-2.69)	<0.001	2.60 (1.62-4.16)	<0.001	1.65 (0.98-2.79)	0.062
ALT								
Normal (2-40)	Ref.		Ref.		Ref.			
Abnormal (<2 or >40)	2.63 (2.28-3.04)	<0.001	1.06 (0.85-1.32)	0.603	1.53 (0.97-2.39)	0.065		
Bili Total								
Normal (0.2-1.2)	Ref.				Ref.			
Abnormal (<0.2 or >1.2)	1.56 (1.31-1.85)	<0.001			1.56 (0.94-2.58)	0.083		
Albumin								
Normal (3.5-5.2)	Ref.				Ref.			
Abnormal (<3.5 or >5.2)	1.68 (1.40-2.00)	<0.001			1.80 (1.06-3.03)	0.029		
HBsAg								
Negative	Ref.				Ref.			
Positive	1.44 (0.82-2.55)	0.206			0.65 (0.09-83.24)	0.774 ^a^		
HBeAg								
Negative	Ref.				Ref.			
Positive	1.08 (0.88-1.34)	0.456			1.05 (0.50-2.21)	0.890		
HBV_DNA								
≤ 20	Ref.							
> 20- ≤ 2000	0.96 (0.70-1.31)	0.791						
> 2000	1.27 (0.99-1.63)	0.059						
AFP								
< 400	Ref.		Ref.		Ref.			
≥ 400	26.73 (20.08-35.58)	<0.001	11.72 (8.16-16.84)	<0.001	2.59 (0.36-18.63)	0.345		
FIB-4 scores (1)								
< 1.3	Ref.		Ref.		Ref.		Ref.	
≥ 1.3	4.10 (3.07-5.49)	<0.001	0.93 (0.66-1.31)	0.696	3.42 (1.38-8.49)	0.008	0.93 (0.36-2.38)	0.873

Abbreviation: DM: diabetes mellitus. PT: prothrombin time. eGFR: estimated Glomerular filtration rate. AST: aspartate aminotransferase. ALT: alanine aminotransferase. Bili Total: Total Bilirubin. HBsAg: hepatitis B virus s antigen. HBeAg: hepatitis B virus e antigen. AFP: alpha-fetoprotein. FIB-4 score: Fibrosis-4 score. ^a^ Cox Regression with Firth's Penalized Likelihood. Because the three variables (PT, Bili Total, and Albumin) have too much missing values, these three variables are not included in the multivariable analysis. Due to the original stratification of FIB-4 scores, there are too few people in some strata. Therefore, it is replaced by FIB-4 score (1).
