# Supplementary material for: Risk Factors and Nomogram Model for Hepatocellular Carcinoma Development in Chronic Hepatitis B Patients with Low-Level Viremia
Source: Int J Med Sci. 2024 Jun 17;21(9):1661–71. doi: 10.7150/ijms.95861 (PMC11241087; doi:10.7150/ijms.95861)
Supplement: Supplementary file 1 — Supplementary tables. [file ijmsv21p1661s1.pdf]

Supplementary Table 1. **The detailed reimbursement guidelines for nucleos(t)ide analogs therapy by the National Health Insurance Service in Taiwan.**

Clinical status	Reimbursement criteria for nucleos(t)ide analogs therapy in CHB patients
Decompensated	PT≥3sec or Total bilirubin≥2mg/dL
Cirrhosis or HCC	Serum HBV DNA≥2,000 IU/mL
HBeAg-positive	Serum ALT≥200 U/mL or ALT≥80 but <200 U/Ml and serum HBV DNA≥20,000 IU/mL
HBeAg-negative	Serum HBV DNA≥2,000 IU/mL and serum ALT≥80 U/Ml more than twice a half year (with an interval of 3 months each time)

Abbreviation: HBeAg: hepatitis B e antigen. CHB: chronic hepatitis B. HBV: hepatitis B virus. AST: aspartate aminotransferase. ALT: alanine aminotransferase. HCC: hepatocellular carcinoma.

Supplementary Table 2. 3-year, 5-year, and 10-year cumulative incidence of hepatocellular carcinoma (life table method).

	MVR (N=1285)	LLV (N=1351)	HBV DNA>2000 (N=3027)	
HCC incidence	%	%	%	p-value [a]
3y	3.64	3.56	4.38	0.349
5y	4.98	4.96	6.68	0.044 [b]
10y	10.54	9.51	12.37	0.034 [c]

Abbreviation: LLV: low-level viremia. MVR: maintained virological response. HBV: hepatitis B virus. HCC: hepatocellular carcinoma.

[a] Log-rank test.

[b] Post-hoc test with Scheffe's method: HBV-DNA >2000 vs. MVR (p-value: 0.075); LLV vs. MVR (p-value: 0.999); HBV-DNA >2000 vs. LLV (p-value: 0.076)

[c] Post-hoc test with Scheffe's method: HBV-DNA >2000 vs. MVR (p-value: 0.078); LLV vs. MVR (p-value: 0.970); HBV-DNA >2000 vs. LLV (p-value: 0.049)

Supplementary Table 3. Score comparison table for predicting the risk of hepatocellular carcinoma in patients with low level viremia.

Total Points	3 year HCC-free probability
42	0.99
117	0.90
141	0.80
156	0.70
168	0.60

Total Points	5 year HCC-free probability
31	0.99
106	0.90
130	0.80
145	0.70
157	0.60
166	0.50

Total Points	10 year HCC-free probability
81	0.9
105	0.80
120	0.70
131	0.60
141	0.50
150	0.40
159	0.30
168	0.20

Abbreviation: HCC: hepatocellular carcinoma.
